# Characterization of a Novel Bacteriophage Henu2 and Evaluation of the Synergistic Antibacterial Activity of Phage-Antibiotics

**DOI:** 10.3390/antibiotics10020174

**Published:** 2021-02-09

**Authors:** Xianghui Li, Tongxin Hu, Jiacun Wei, Yuhua He, Abualgasim Elgaili Abdalla, Guoying Wang, Yanzhang Li, Tieshan Teng

**Affiliations:** 1 Institute of Biomedical Informatics, School of Basic Medical Sciences, Henan University, Kaifeng 475004, China; 10190158@vip.henu.edu.cn (X.L.); 1819010169@vip.henu.edu.cn (T.H.); 1819010208@vip.henu.edu.cn (J.W.); 1819010160@vip.henu.edu.cn (Y.H.); 10190115@vip.henu.edu.cn (Y.L.); 2 Department of Clinical Laboratory Sciences, College of Applied Medical Sciences, Jouf University, Sakaka 2014, Saudi Arabia; aealseddig@ju.edu.sa

**Keywords:** *Staphylococcus aureus*, bacteriophage, phage-antibiotic synergy

## Abstract

*Staphylococcus aureus* phage Henu2 was isolated from a sewage sample collected in Kaifeng, China, in 2017. In this study, Henu2, a linear double-stranded DNA virus, was sequenced and found to be 43,513 bp long with 35% G + C content and 63 putative open reading frames (ORFs). Phage Henu2 belongs to the family *Siphoviridae* and possesses an isometric head (63 nm in diameter). The latent time and burst size of Henu2 were approximately 20 min and 7.8 plaque forming unit (PFU)/infected cells. The Henu2 maintained infectivity over a wide range of temperature (10–60 °C) and pH values (4–12). Phylogenetic and comparative genomic analyses indicate that *Staphylococcus aureus* phage Henu2 should be a new member of the family of *Siphoviridae* class-II. In this paper, Phage Henu2 alone exhibited weak inhibitory activity on the growth of *S. aureus*. However, the combination of phage Henu2 and some antibiotics or oxides could effectively inhibit the growth of *S. aureus*, with a decrease of more than three logs within 24 h in vitro. These results provide useful information that phage Henu2 can be combined with antibiotics to increase the production of phage Henu2 and thus enhance the efficacy of bacterial killing.

## 1. Introduction

*Staphylococcus aureus* is a Gram-positive cocci bacterium commonly isolated from the skin and nostrils of approximately one-third of the global population [1]. It is the leading cause of wound infection ranging from mild skin infection to severe soft tissue infection potentially resulting in severe metastatic infection [2]. Since the 1940s, *S. aureus* has developed resistance to numerous antibiotics in clinical use. The bacterium employs multiple strategies to escape from antibiotics including deactivation of antibiotics by releasing enzymes including β-lactamases and shifting of drug-target such as alteration of penicillin-binding proteins (PBP) into PBP2a, a major mechanism of resistance to β-lactam antibiotics [3,4]. An increasing prevalence of methicillin-resistant *S. aureus* (MRSA) is one of the major global public health concerns [5,6]. Dissemination of MRSA with multi-resistance phenotypes has significantly caused limited treatment options of *S. aureus* infections [7,8]. As such, it is urgently essential to explore non-antibiotic approaches for the treatment of *S. aureus* infections.

Bacteriophages are ancient enemies of bacteria shown to exhibit natural antibacterial benefits [9,10,11]. They are extremely ubiquitous in nature, with an estimated order of 10^31^ [12], demonstrating clinical potential as therapeutic agents for the topical or non-systemic treatment of bacterial infections [13]. Despite their therapeutic potential major shortcomings remain on the emergence of bacteriophage resistance. Recent novel approaches have been proposed to resolve the main problem of using phages, including phage cocktails and phage-antibiotic combinations [14,15]. Despite many studies showing that phage cocktail therapy exhibits an effective antibacterial effect and prevents the emergence of phage resistance, the single bactericidal mechanism and the need for large-scale screening of different bacteriophages have limited its application [16,17,18,19]. Notably, phage-antibiotic synergy (PAS) is a phenomenon where a sub-inhibitory concentration of antibiotics enhances phage induced host cell decline. Due to their different bactericidal mechanisms and the limitation in the evolution of common resistance, (PAS) has shown a better bactericidal activity [20,21,22]. At the same time, phage-antibiotic synergy is used to combat drug-resistant bacteria, including multidrug-resistant, extensively-drug resistant, or pan drug-resistant bacteria [23,24,25,26]. Moreover, PAS has shown potential in different bacteria, including *Pseudomonas aeruginosa*, *Escherichia. coli*, *Klebsiella. Pneumoniae*, and *Burkholderia cepacian* by different phage-antibiotic combinations [27,28,29,30,31,32,33]. Limited reports have confirmed the synergistic effect of phages and antibiotics against *S. aureus* [34,35,36,37,38]. Nonetheless, existing data are scarce as well as inconsistent, and the activity of important groups of antibiotics remains underexplored in combination with *S. aureus* phages [39]. As such, this study evaluated the efficacy of subinhibitory concentrations of antibiotics (Clarithromycin, Linezolid, Cefotaxime, Tetracycline and Ciprofloxacin) and hydrogen peroxide combined with the phage Henu2 from the *Siphoviridae* family geared towards examining the ability of phage-antibiotic synergy in the killing of *S. aureus*.

## 2. Results

### 2.1. Isolation and Morphology

**Figure 1:**
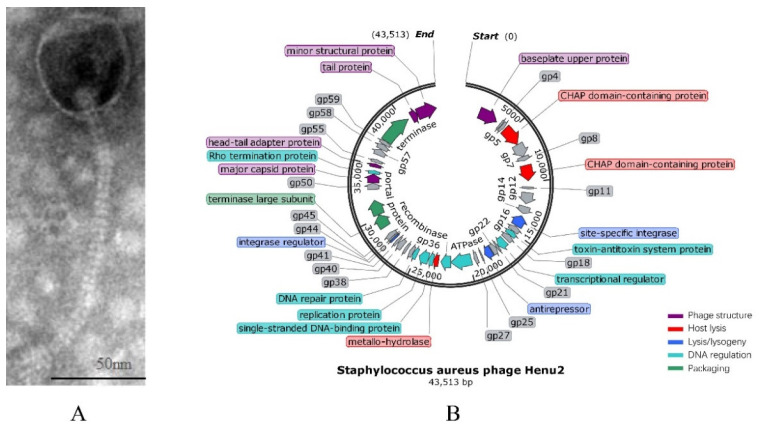
(**A**) Transmission electron micrograph of negatively stained Henu2 phage particles; (**B**) Genomic map of *Staphylococcus aureus* phage Henu2: Sixty-three predicted open reading frames (ORFs) are represented by different colored blocks.

Phage Henu2 was isolated from sewage water using *S. aureus* N315 as a host [40]. Henu2 has an isometric head with a width of 63nm in diameter and a tail with a length of 186 nm long (Figure 1A). The baseplate structure of the phage Henu2 comprised multiple disc baseplates similar to that of phage SA97 [41]. Notably, Henu2 belongs to the genus *Phietavirus* in the family of *Siphoviridae* [42].

### 2.2. General Genome Analysis

The genomic DNA of Henu2 was extracted as described in the Section 4. The gene map illustrated tightly packed coding regions, with significantly few intergenic spaces between them. The complete genome sequence of Henu2 had a linear double-stranded DNA molecule of 43,513 bp with 35% G + C content. The gene-coding potential of Henu2 is 92.2% with 1.46 genes per kilobase pair of nucleotide sequence, a number similar to that reported for *S. aureus* bacteriophage 66 (1.48 genes per kbp) [43]. Additionally, it was found that Henu2 phage belongs to class-II (about 40 kbp) based on genomic size (Table S1).

### 2.3. Biological Characteristics of Henu2 Phage

**Figure 2:**
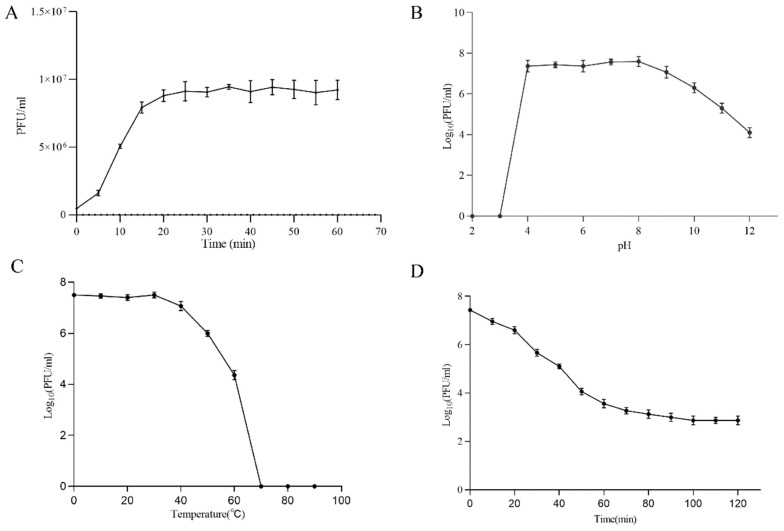
(**A**) One-step growth experiment; (**B**) pH stability test; (**C**) Thermal stability test; (**D**) and UV sensitivity of phage Henu2.

To determine the optimal MOI, the phage Henu2 was incubated with *S. aureus* strain N315 with five MOI values of 0.001, 0.01, 0.1, 1 and 10, respectively. Based on the findings in Table 1, the phage titer reached 6 × 10^8^ PFU/mL when MOI = 0.1 was the highest titer among the above five MOI values. Therefore, the optimal MOI of phage Henu2 was 0.1. According to the results shown in Figure 2A (Table S4), Henu2 has a latent period of approximately 20 min and a burst size of approximately 7.8 PFU per infected cell.

Furthermore, Henu2 infectivity towards *S. aureus* remained unaffected by chloroform, indicating that Henu2 was not a lipid-containing phage. Phage Henu2 retained its infectivity toward *S. aureus* when incubated at 50 °C or lower, indicating satisfactory thermal stability. Nevertheless, the titer of Henu2 gradually decreased when incubated at a temperature higher than 50 °C (Figure 2C, Table S6). Phage Henu2 was stable for 1h at pH values from 4 to 11. Almost no viable phages were observed at pH values 2–3. These results suggest that extreme pH potentially affect phage Henu2 infectivity (Figure 2B, Table S5). The UV sensitivity test of phage Henu2 showed that the titer of Henu2 had a significant reduction in the first 60 min, then slowly reduced at 40 min and stabilized at 100 min (Figure 2D, Table S7).

### 2.4. Comparative Genome Analysis

**Figure 3:**
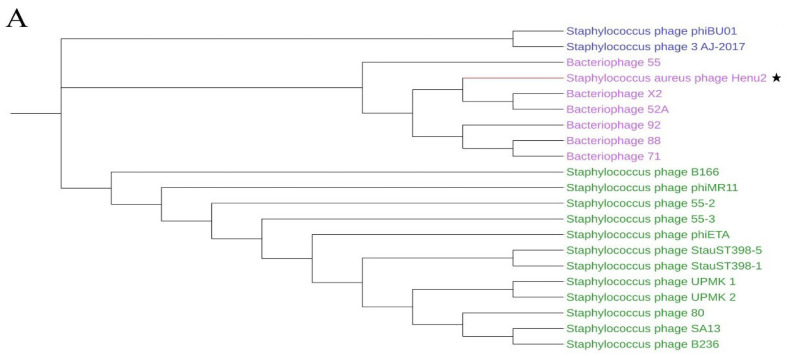

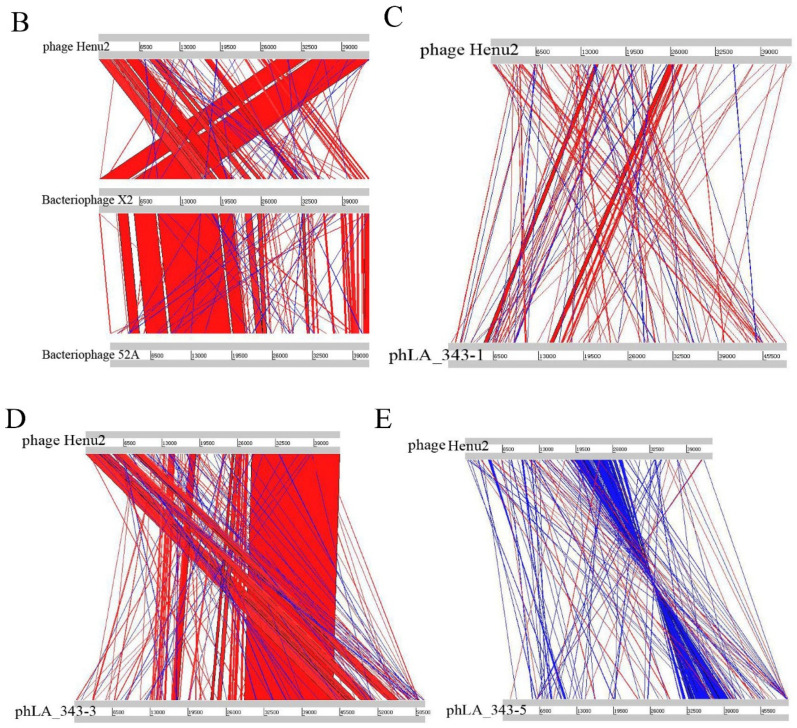
(**A**) Phylogenetic analysis of various phages that infect *Staphylococcus aureus*. The tree was drawn by MEGA7 program using the NJ method based on the gene encoding terminase; (**B**) comparative genome analysis of phage Henu2, bacteriophage 52A and Bacteriophage X2. Comparisons were performed using the BLASTn and ACT (Artemis Comparison Tool) programs; (**C**–**E**) comparative genome analysis of phage Henu2, bacteriophage LA_343-1, bacteriophage LA_343-3 and bacteriophage LA_343-5. Red and blue lanes connecting genomes represent syntenic blocks in direct and reverse orientations, respectively.

A total of 20 Staphylococcal phages showed similarity with Henu2 based on BLASTn (Table S2). The genome length of all the phages listed in Table S2 was about 40 k bp except phage UPMK_1. The total of phage had a high identity with Henu2 (≥92%), but low query cover (≤63%), particularly for phiBU01 and 3-AJ-2017 (19% and 20%, respectively). Phylogenetic analysis of Henu2 based on large-subunit of terminase indicated that Henu2 was most similar to the phage 52A and X2. Meanwhile, comparative genome analysis was performed using Artemis Comparison Tool (ACT) among phage Henu2, 52A and X2. As shown in Figure 3B, the genes implicated in the structure and assembly of phages were highly similar in all three genomes.

Interestingly, upon BLASTn analysis of Henu2, several prophage genomes integrated into *S. aureus* genome were observed in the results of BLASTn analysis. One *S. aureus* strain (*S. aureus* isolate 17_LA_343, Accession: LT992471) was predicted to contain five prophages, three genomes of which were intact (Table 2). Furthermore, comparative genome analysis was performed among Henu2 and three intact prophages as shown in Figure 3C–E.

### 2.5. Host Range of Phage Henu2

The host range of phage Henu2 was analyzed as spot test assay and relative efficiency of plating (REOP) for 41 *S. aureus* strains and 9 other strains as shown in Table 3 [44]. Henu2 phage demonstrated a wide host range infecting 34 out of 41 *S. aureus* strains, including MRSA and MSSA strains. Interestingly, that the infectivity of Henu2 did not depend on drugs susceptibility patterns of *S. aureus* strains. A total of nine non-*S. aureus* strains were resistant to Henu2 phage infection. These results indicate that Henu2 specifically targets *S. aureus* [45].

### 2.6. Effect of Sub-Lethal Antibiotics on Adsorption and Burst Size of Phage Henu2

**Figure 4:**
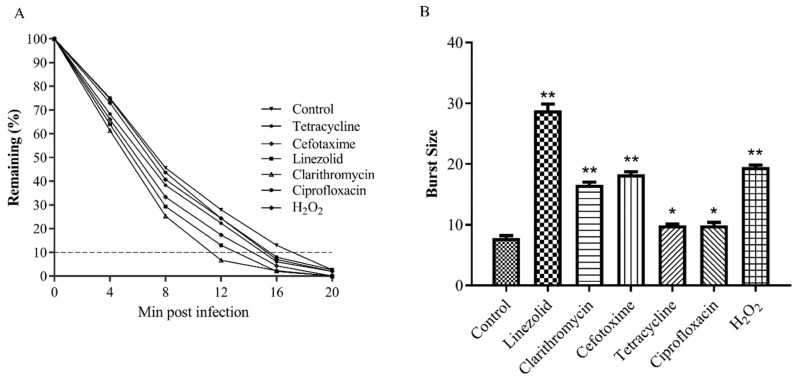
(**A**) Adsorption of phage Henu2 to *S. aureus* (MOI = 0.1) in the presence of sub-lethal concentrations of various antibiotics (*p* < 0.01), 90% phage adsorbed to host bacteria is used as cutoff; (**B**) Burst sizes measured in the presence of antibiotics inducing PAS (*, *p* < 0.05; **, *p* < 0.01).

PAS is a phenomenon where the sub-lethal concentration of a few antibiotics significantly stimulates the generation of progeny phages in host bacterial cells [21]. The MIC values of the antibiotics used in this study are shown in Table S3. The growth characteristics of phage Henu2, including phage absorption rate and burst size, were used to detect whether phage Henu2 had a synergistic effect with sub-lethal concentrations of antibiotics. The results shown in Figure 4A indicate the total time required for phage adsorption in the presence of five different antibiotics and hydrogen peroxide. The findings revealed 20 min as the average adsorption time of phage Henu2 on the surface of *S. aureus*. Clarithromycin and linezolid bound to the 50S ribosome and inhibited bacterial protein synthesis causing a reduction of the total adsorption time of Henu2 from 20 min to 12 min and 14 min, respectively. Similarly, in the presence of Cefotaxime, an inhibitor of bacterial cell wall synthesis, the adsorption time significantly shortened to 16 min. In the case of tetracycline and ciprofloxacin, a significant decrease in the time required for 80% adsorption (12 min and 16 min, respectively) was observed. Interestingly, Henu2 had an adsorption time of 15 min with hydrogen peroxide.

At 20 min post-infection in the absence of any antibiotics, the decrease of the numbers of phage Henu2 was stopped in culture medium, indicating the occurrence of bacterial burst (Figure 4A, Table S8). The average burst size was 7.8 phages released per host cell, with a phage absorption period of 20 min. In contrast, clarithromycin and linezolid significantly increased the burst size, to an average value of 16.6 and 28.8 phage/cell (*p* < 0.01). Similarly, an increase in the burst size was from 7.8 to 18.3 phage/cell. (*p* < 0.05) was observed with Cefotaxime as well. Tetracycline and ciprofloxacin were nearly comparable with each other, significantly increasing the burst size by 9.9 phage/cell. Nonetheless, in the presence of a sub-lethal dose of hydrogen peroxide, the increase in burst size was 2.5-fold (19.5 phage/cell) (Figure 4B, Table S9).

### 2.7. Time–Kill Analyses for Assessing the PAS Efficacy

**Figure 5:**
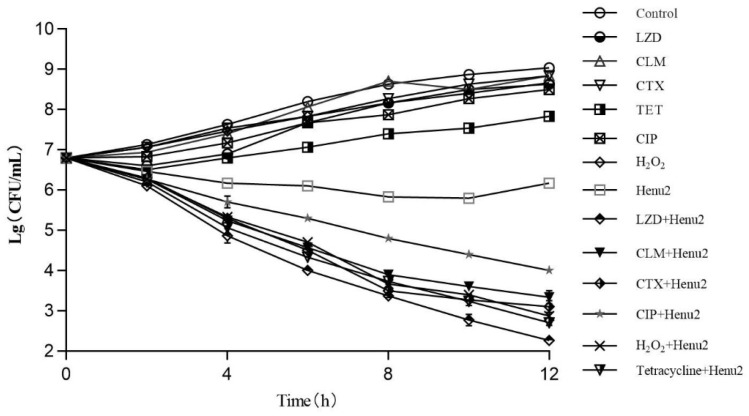
Killing effect of phage Henu2 on *S. aureus* N315 logarithmic phase cells in the presence of linezolid (LZD) *, clarithromycin (CLM) *, cefotaxime (CTX) *, ciprofloxacin (CIP) *, tetracycline (TET) * and hydrogen peroxide *; Cells were treated with 2.25 μg/mL linezolid, 4.1 μg/mL clarithromycin, 16 μg/mL Cefotaxime, 1.25 μg/mL ciprofloxacin, 7 μg/mL tetracycline or 0.25% hydrogen peroxide. Phage Henu2 was added at a MOI of 0.1 Values given are averages standard deviations from three replicates (*, *p* < 0.01).

The antibacterial effect of phages, antibiotic and phage/antibiotic combination was assessed against *S. aureus* at 37 °C as illustrated in Figure 5 (Table S10). The cell number of *S. aureus* was increased by 0.6–1.8 logs over 12 h in the presence of sub-inhibitory concentrations of five different antibiotics and hydrogen peroxide alone. After treatment with phage Henu2 alone, the number of *S. aureus* continuously decreased up to 10 h then steadily increased. In contrast, the combination treatment of phage Henu2 and antibiotics, including clarithromycin, linezolid, cefotaxime, tetracycline, ciprofloxacin and hydrogen peroxide, significantly inhibited the growth of *S. aureus* up to the early 12 h of incubation, causing 2.6–4.4 logs reductions. These results verify that phage Henu2 combined with the sub-inhibitory concentrations antibiotics effectively decreased the cell concentrations of *S. aureus.*

## 3. Discussion

*S. aureus* infecting phage Henu2 was isolated, intensely characterized with phenotypes and genomes, and analyzed for antimicrobial activity. Based on genome sequence similarity, Henu2 was classified as a member of the genus *Phietavirus* in the family of *Siphoviridae* [46]. High genetic similarity between Henu2 and prophage suggested that Henu2 might be originating from the prophage embedded in the genome of *S. aureus* isolate 17_LA_343. The prophage genome is often integrated into the bacterial chromosome as a lysogenic cycle of temperate phage [47,48]. The repressor and anti-repressor proteins were responsible for the switch of the lytic and lysogenic cycle of prophage. In the Henu2 genome, one anti-repressor protein was detected, but not the repressor. Hatful et al. showed that most of the two proteins encoding gene (NTRK3) in the lytic phage were mutated in the evolutionary process [49]. Based on this, it is tempting to speculate that Henu2 might be transformed into a lytic phage by losing the inhibitor protein during the evolution process.

This paper used *S. aureus* N315 as a host to evaluate the biological characteristics and bactericidal activity of phage Henu2. Nonetheless, using PHASTER online tool, two prophages were presented in the genome of *S. aureus* N315, one which was an intact prophage with 47 kb length and the other was questionable with 19.9 kb length according to the criteria for scoring prophage regions (as intact, questionable, or incomplete) [50,51]. However, both prophages genomes contained enterotoxin coding genes. As previously mentioned, many factors influence the induction of prophages including antibiotics, UV, temperature or H_2_O_2_ that are well documented [52,53]. Thus, further studies are necessary to establish either or not antibacterial and H_2_O_2_ induce the production of endotoxin in host bacteria.

A comprehensive mechanism of plaque formation remains unknown to scholars. Despite many factors influencing this process, only a few factors have been extensively studied. Therefore, the problems on plaque morphology and size are common in laboratory practice. Based on previous reports, two widely recognized factors were considered responsible for the enlarged plaques, including filamentation of bacteria and the delayed lysis of bacteria. Filamentation of cells produced by antibiotics or other external factors increased its surface area, thereby promoting more phage adsorption and the generation of progeny phage. The phenomenon of delayed lysis of bacteria, caused by the effect of sublethal concentration antibiotics, influenced the lysis period of bacteria and increased the number of phage copies before the lysis of bacteria [36]. Using the double-layer agar plate (DLAP) method, we found that the plaque formed by phage Henu2 had no difference with the antibiotic treatment group. ŁOS’ et al. demonstrated that the size and morphology of plaques were influenced by several factors, including the types of carbon sources in the medium, the selected strains as host bacteria, the number of host bacteria, the types of antibiotics and the thickness of the upper agar, etc. [14,54]. Meanwhile, the burst size and latent period of phages were significantly affected by these factors. As such, further research should be conducted to investigate the effect of different detection methods on phage production.

Moreover, the complex physiological environment showed a significant effect on the activity of phages combined with antibiotics. The undesired side-effects and beneficial effects of phage infection in vivo include. First, based on the latest reports, the observed synergism effect of phages with antibiotics might be decreased when the culture conditions were changed into urine or blood similar to the physiological environment of the human body [55,56]. Furthermore, the host bacteria degraded by phages produced many proteins, lipids, endotoxin and cell fragments, released into the human internal environment often causing inflammation. Finally, a few antibiotics might stimulate the expression of virulence genes, indicating that the type of antibiotics should be selected to prevent over-generation of virulence products [57]. However, phages also demonstrated profound effects on the outcome of bacterial infections by modulating the immune response. Reports indicate that immune cells, including macrophages and neutrophils, also have a synergistic effect with phages in vivo, known as immune-phage synergy [58]. Although the specific mechanism needs to be addressed, studies have shown that phages improve the phagocytic capacity of macrophages [59,60], and preferentially enhance the elimination of phage-resistant bacteria by neutrophils [61,62,63,64].

In conclusion, despite phage potentially having synergistic effects with antibiotics in vitro and in vivo [61,65], several issues should be first resolved for its clinical application and a greater focus on the decrease of the possible harm to humans warrants investigation in further research.

## 4. Materials and Methods

### 4.1. Isolation and Purification of S. aureus Phage

*S. aureus* N315, a drug-resistance strain, was stored in our lab and used as a host. Bacteriophage Henu2 was isolated from sewage water obtained from Campus Lake in Henan University using the double-layer agar method as previously described by Pajunen et al. [66]. Briefly, 10 mL sewage samples were mixed with 10 mL Luria–Bertani (LB) medium and 1 ml *S. aureus* N315, then co-incubated in conical flasks at 30 °C with shaking in 180 rpm for 48 h. The culture was filtered using a 0.22 μm membrane filter and the filtration product was serially diluted to detect phage activity using a double-layer agar plate. A single plaque was picked up from the double-layer agar plate using a sterile inoculation loop then serially purified 6 times. The TE buffer (13.6 mM NaCl, 2 mM Tris, 2 mM MgCl_2_ and 0.01% gelatin, pH 7.5) was used to prepare the stock cultures of Henu2.

### 4.2. Electron Microscopy

The morphology of Henu2 was identified via transmission electron microscopy. Phage particles were purified by centrifugation at 4 °C with a speed of 25,000 g for 1 h using a Beckman J2–21 centrifuge with a JA-18.1 fixed rotor. The purified phage was adsorbed to carbon-coated copper grids, fixed with glutaraldehyde (2.5% *v*/*v*) and negatively stained with 3% uranyl acetate. After drying, grids were examined under a transmission electron microscope JEM-1400 at 200 kV.

### 4.3. Phage DNA Isolation

Extraction of phage DNA was performed using chloroform-standard phenol extraction method as previously described [67] with a few modifications. To remove free DNA and RNA, DNase I and RNase with a final concentration of 5 μg/mL and 1 μg/mL, respectively, were incubated with the sample at 37 °C for 1 h. Then, Tris-Saturated Phenol was used to extract nucleic acid from the solution and this step was repeated twice. The sample was precipitated with the 1/10 sodium acetate (*v*/*v*) and 1/1 absolute ethanol (*v*/*v*), respectively. Then, the above solution was stored at −20 °C overnight then centrifuged to remove the supernatant. The precipitate was washed twice with absolute and 70% ethanol. The quantity and purity of DNA extracted was spectrophotometrically investigated using a Nanodrop.

### 4.4. Genome Sequencing and Termini Identification

The complete genome was sequenced using 454 technology with the GS Junior 454 system platform (Roche Applied Science, Indianapolis, IN, USA), yielding 6.5-fold coverage of the phage genome [68]. Prediction of open reading frames (ORFs) was performed using softberry (http://linux1.softberry.com/ (accessed on 8 February 2021)) and Glimmer. A whole-genome sequence assembly was performed using Geneious software version 6 while Phage termini were identified using the method described by Zhang et al. [69].

### 4.5. The Multiplicity of Infection (MOI) of Phage Henu2

To determine the optimal MOI, the logarithmic phase *S. aureus* and a serial dilution of Henu2 were co-incubated in 1.5 mL polypropylene tubes. The ratio of bacteriophage and *S. aureus* was adjusted to 10, 1, 0.1, 0.01 and 0.001 respectively. Then, the mixture was cultured in a shaking incubator at 30 °C, for 4 h. Subsequently, the mixture was centrifuged at 10,000 rpm for 1 min and filtered to remove the host bacteria. Then the bacteriophage titer was determined using the double-layer agar method. The mixture with a ratio of Henu2 to *S. aureus* with the highest phage titer value was considered the optimal multiplicity of infection (OMOI). A total of 3 replicates were performed.

### 4.6. One-Step Growth Curve of Phage Henu2

*S. aureus* was infected with phage Henu2 at an MOI of 0.1 then incubated at 30 °C for 10 min. The mixture was then centrifuged at 10,000 rpm for 1 min to remove unabsorbed phage particles. The cell pellet was quickly re-suspended in 1mL of fresh Luria–Bertani (LB) medium. Subsequently, the suspension was added to 5mL of LB broth and incubated in a shaking incubator at 30 °C. Meanwhile, the sample was collected at intervals of every 5 min for one hour. The phage titer was determined by the double-layer agar plate method. A total of 3 replicates were performed.

### 4.7. Thermal, pH, and Chloroform Stability Tests

The phage suspensions in normal saline with a titer of 5 × 10^7^ were incubated at various temperatures (0 to 80 °C) and aliquot (100 μL) were collected after 1 h, respectively. Then, the double-layer agar and spot test were used to evaluate the thermal stability of Henu2. To evaluate the pH stability of the Henu2 phage, the pH of the phage buffer was adjusted in wide range from 2 to 12. After incubating at 30 °C for 1 h, the amount of phage Henu2 was diluted and two-layer agar method was used to count the phage titer. To test the effect of chloroform, 10 μL chloroform was added to 1ml of Henu2 suspension with a titer of 1 × 10^6^. The solution was gently mixed and incubated for 1 h at room temperature. Then, the phage titer was determined by the double agar-layer agar method. Phage Henu2 was exposed to UV at 50 J/m^2^. The titer was determined every 10 min for 2 h using the double agar-layer agar method. A total of 3 replicates were performed.

### 4.8. The MIC of the Host Bacterial

To establish the minimum inhibitory concentrations (MIC) of antibiotics, 900 μL logarithmic phase host bacteria and 100 μL antibiotics with different concentrations (clarithromycin, linezolid, cefotaxime, tetracycline and ciprofloxacin) were mixed into a 1.5 mL polypropylene tubes, then the 100 μL mixture was removed into a 96-wells plant and cultured at 30 °C for 24 h. The minimum concentration of antibiotics inhibiting the growth of *S. aureus* was considered MIC value. A total of 3 replicates were performed.

### 4.9. Bioinformatic Analysis

BlastP and BlastN in NCBI (https://blast.ncbi.nlm.nih.gov/Blast.cgi (accessed on 8 February 2021)) were used to compare the known Henu2 protein sequence and nucleic acid sequence with protein library and nucleic acid library, respectively. The phylogenetic tree was constructed by MEGA7 software and iTOL (https://itol.embl.de (accessed on 8 February 2021)) [70]. Phage with the greatest similarity to Henu2 was identified by phylogenetic tree, while complete genome sequence alignment, was conducted using a double ACT (http://www.hpa-bioinfotools.org.uk/pise/double_actv2.html (accessed on 8 February 2021)) online program. The functional proteins were indicated by different colors, while the hypothetical proteins were marked with yellow. Based on the GenBank information, a web-based tool called SnapGene was used for making a map of Henu2 genomes [71,72].

### 4.10. Host Range Analysis

Phage host range was established using a spot test and efficiency-of-plating (EOP) method combined with double-layer agar plate method following a previously described protocol [73,74]. All the tested strains listed in Table 3 were cultured for 16 h at 30 °C. Then, 150 µL of the bacterial culture of each strain was added to 3 mL of the 0.8% soft agar and overlaid on the LB or Brain Heart Infusion (BHI) agar plate. Phage titer of Henu2 was adjusted to 10^6^ PFU with phage buffer, and 5 µL of phage suspension was spotted onto the surface of double-layer agar plate then incubated at 30 °C for 36 h and the plaque formation was examined at every 8 h. For the efficiency-of-plating (EOP) method, 10 µL phage was mixed with 150 µL each strain bacterial culture and incubated at 30 °C for 5 min. Then, the mixture added to 3ml efficiency of plating (EOP) was calculated by dividing the phage titer on the test strain by the phage titer on the reference strain. A total of 3 replicates were performed.

## Figures and Tables

**Table 1:** Optimal multiplicity of infection (MOI) of bacteriophage Henu2.

Phage Titer Log_10_ (PFU/mL)	Bacterial Titer Log_10_ (CFU/mL)	MOI	Mean ± SD
7.78	6.78	10	8.26 ± 0.20
6.78	6.78	1	6.84 ± 0.05
5.78	6.78	0.1	8.77 ± 0.06
4.78	6.78	0.01	6.69 ± 0.07
3.78	6.78	0.001	5.59 ± 0.09

**Table 2:** Summary of genomics features of prophage in Staphylococcus *aureus* isolate 17_LA_343.

Prophage Name	Region Length	Completeness	CDS Number	Region Position	GC Content
phLA_343-1	48.8 Kbp	Intact	71	389,425–438,317	33.71%
phLA_343-2	16.4 Kbp	Incomplete	17	502,332–518,743	31.96%
phLA_343-3	60 Kbp	Intact	75	924,419–984,468	35.49%
phLA_343-4	22 Kbp	Incomplete	10	2,129,407–2,151,503	28.98%
phLA_343-5	50.3 Kbp	Intact	71	2,147,904–2,198,245	32.66%

**Table 3:** Host range analysis of phage Henu2.

No.	Strains	Source	Spot Assay	REOP
1	*S. aureus* AB91118 (MSSA)	CCTCC	+	1
2	*S. aureus* KFS1001 (MSSA)	Human	−	−
3	*S. aureus* KFS1002 (MSSA)	Human	+	0.86
4	*S. aureus* KFS1003 (MSSA)	Human	+	0.08
5	*S. aureus* KFS1006 (MSSA)	Human	+	0.13
6	*S. aureus* KFS1007 (MSSA)	Human	−	0
7	*S. aureus* KFS1008 (MSSA)	Human	−	0
8	*S. aureus* KFS1009 (MSSA)	Human	+	0.34
9	*S. aureus* KFS1010 (MSSA)	Human	+	0.26
10	*S. aureus* KFS1013 (MSSA)	Human	+	0.24
11	*S. aureus* KFS1014 (MSSA)	Human	+	0.32
12	*S. aureus* KFS1015 (MSSA)	Human	+	0.12
13	*S. aureus* KFS1016 (MSSA)	Human	+	0.66
14	*S. aureus* KFS1017 (MSSA)	Human	+	0.21
15	*S. aureus* KFS1018 (MSSA)	Human	+	1.03
16	*S. aureus* KFS1027 (MSSA)	Human	+	0.42
17	*S. aureus* KFS1028 (MSSA)	Human	+	0.05
18	*S. aureus* KFS1029 (MSSA)	Human	+	0.13
19	*S. aureus* KFS1030 (MSSA)	Milk	+	0.42
20	*S. aureus* KFS1031 (MSSA)	Milk	+	0.04
21	*S. aureus* KFS1032 (MSSA)	Milk	+	0.37
22	*S. aureus* KFS1033 (MSSA)	Milk	+	0.02
23	*S. aureus* KFS1034 (MSSA)	Livestock	+	0.75
24	*S. aureus* KFS1035 (MSSA)	Livestock	+	0.17
25	*S. aureus* KFS1036 (MSSA)	Livestock	+	0.09
26	*S. aureus* KFS1037 (MSSA)	Livestock	+	0.36
27	*S. aureus* KFS1038 (MSSA)	Livestock	+	0.93
28	*S. aureus* KFS1039 (MSSA)	Livestock	+	0.50
29	*S. aureus* N315 (MRSA)	Human	+	0.50
30	*S. aureus* AM001 (MRSA)	Human	+	0.10
31	*S. aureus* AM002 (MRSA)	Human	+	0.33
32	*S. aureus* AM005 (MRSA)	Human	−	0
33	*S. aureus* AM006 (MRSA)	Human	−	0
34	*S. aureus* AM007 (MRSA)	Human	+	0.25
35	*S. aureus* AM008 (MRSA)	Human	−	0
36	*S. aureus* AM010 (MRSA)	Human	+	0.38
37	*S. aureus* AM018(MRSA)	Human	+	0.03
38	*S. aureus* AM022 (MRSA)	Human	+	0.09
39	*S. aureus* AM023 (MRSA)	Human	+	0.21
40	*S. aureus* AM024 (MRSA)	Human	−	0
41	*S. aureus* AM025 (MRSA)	Human	−	0
42	*L. monocytogenes* 19115	ATCC	−	0
44	*B. thuringiensis* 1765	Lab storage	−	0
45	*B. cereus* IS195	Lab storage	−	0
46	*E. faecium* 35667	ATCC	−	0
47	*E. coli* BL21(DE3)	Lab storage	−	0
48	*S. dysgalactiae* 35666	ATCC	−	0
49	*S. albus* 8799	ATCC	−	0
50	*B. cereus* 33018R	ATCC	−	0
51	*S. pyogenes* 12344	ATCC	−	0

MSSA, methicillin-susceptible *S. aureus*; MRSA, methicillin-resistant *S. aureus*; CCTCC: China Center for Type Culture Collection; ATCC: American Type Culture Collection; REOP: relative efficiency of plating. REOP was determined as ratio of plaque forming unit (PFU) on *S. aureus* AB91118 versus PFU on the control strains; +, clear plaques; −, no plaque.

## Data Availability

Data is contained within the article or supplementary material. Complete genome of *Staphylococcus* phage Henu2 is available in https://www.ncbi.nlm.nih.gov/nuccore/ under the GenBank accession number MK211557.1.

## Supplementary Materials

The following are available online at https://www.mdpi.com/2079-6382/10/2/174/s1, Table S1: Predicted molecular function for gene products of phage Henu2, Table S2: Summary of similar genomic sequence with phage Henu2, Table S3: List of MIC values of antibiotics, Table S4: One-step growth experiment (Mean ± SD), Table S5: pH stability test (Mean ± SD), Table S6: Thermal stability test (Mean ± SD), Table S7: UV sensitivity of phage Henu2 (Mean ± SD), Table S8: Adsorption of phage Henu2 to S. aureus (MOI = 0.1, Mean ± SD), Table S9: Burst sizes measured in the presence of antibiotics inducing PAS (Mean ± SD), Table S10: Killing effect of phage Henu2 on S. aureus N315 logarithmic phase cells (Mean ± SD).
