# Supplementary material for: Characterization of a Novel Bacteriophage Henu2 and Evaluation of the Synergistic Antibacterial Activity of Phage-Antibiotics
Source: Antibiotics (Basel). 2021 Feb 9;10(2):174. doi: 10.3390/antibiotics10020174 (PMC7916345; doi:10.3390/antibiotics10020174)
Supplement: Supplementary file 1 [file antibiotics-10-00174-s001.pdf]

Table S1. Predicted molecular function for gene products of phage Henu2.

Serial number	Start and Stop	Strand	ORF length (bp)	Molecular weight of protein (kDa)	Predicted molecular function	Hit	Coverage (%)	E-Value	Identity (%)	Accession
1	7-1680	+	1674	63.9	hypothetical protein	hypothetical protein	100	0.0	100.00	WP_160177536.1
2	1680-3503	+	1824	67.4	baseplate upper protein	BppU family phage baseplate upper protein	100	0.0	100.00	WP_113613161.1
3	3503-3880	+	378	14.2	DUF2977 domain-containing protein	DUF2977 domain-containing protein	100	2×10^{-84}	100.00	WP_000705914.1
4	3881-4063	+	183	7.26	hypothetical protein	XkdX family protein	100	4×10^{-35}	100.00	WP_000411344.1
5	4104-4403	+	300	11.8	DUF2951 domain-containing protein	DUF2951 domain-containing protein	100	2×10^{-63}	100.00	WP_000466769.1
6	4540-6414	+	1875	71	CHAP domain-containing protein	glucosaminidase domain-containing protein	100	0.0	100.00	WP_138271972.1
7	6427-7665	+	1239	46.1	DUF2479 domain-containing protein	BppU family phage baseplate upper protein	100	0.0	100.00	WP_015984527.1
8	7670-8065	+	396	14.4	hypothetical protein	hypothetical protein	100	1×10^{-87}	100.00	WP_000398857.1
9	8121-8558	+	438	15.7	phage holin	phage phi LC3 family holin	100	6×10^{-101}	100.00	EUK61875.1
10	8512-9984	+	1473	55.1	CHAP domain-containing protein	SH3 domain-containing protein	98	0.0	100.00	WP_138271973.1
11	10547-10747	+	201	32.1	hypothetical protein	hypothetical protein	100	3×10^{-39}	100.00	WP_000382163.1
12	11094-12143	+	1050	40.9	hypothetical protein	hypothetical protein CH52_01300	100	0.0	100.00	AHM68971.1
13	12058-12423	+	366	14.6	hypothetical protein	hypothetical protein V403_02077	100	1×10^{-80}	100.00	EWY25223.1

14	12420-13103	+	684	27	hypothetical protein	hypothetical protein	100	4×10^{-162}	100.00	WP_000180531.1
15	13346-14395	-	1050	40.9	site-specific integrase	site-specific integrase	100	0.0	100.00	WP_000146088.1
16	14457-14978	-	522	19.4	hypothetical protein	hypothetical protein	98	2×10^{-118}	100.00	WP_138271955.1
17	14990-15460	-	471	18.5	toxin-antitoxin system	putative toxin-antitoxin system, toxin component	100	7×10^{-108}	100.00	ADQ77119.1
18	15464-15985	-	522	20.1	hypothetical protein	hypothetical protein	100	3×10^{-25}	99.42	AUM57685.1
19	15930-16166	+	237	9	XRE transcriptional regulator	helix-turn-helix transcriptional regulator	100	7×10^{-50}	100.00	WP_001121027.1
20	16180-16956	+	777	29.6	transcriptional regulator	Rha family transcriptional regulator	100	0.0	100.00	WP_031783556.1
21	16985-17152	+	168	6.4	hypothetical protein	hypothetical protein	100	2×10^{-27}	100.00	WP_162852001.1
22	17203-17685	-	483	18.6	hypothetical protein	hypothetical protein	100	2×10^{-109}	100.00	WP_033858402.1
23	17745-18458	+	714	27.3	Anti-repressor	ORF6C domain-containing protein	100	1×10^{-175}	100.00	WP_054249650.1
24	18471-18680	+	210	8.14	hypothetical protein	hypothetical protein	100	6×10^{-42}	100.00	WP_000455727.1
25	18714-18824	+	111	3.91	hypothetical protein	hypothetical protein StauST398-3_0009	100	6×10^{-15}	94.44	YP_008059825.1
26	18944-19261	+	318	12.9	hypothetical protein	hypothetical protein	100	1×10^{-64}	100.00	WP_000829613.1
27	19194-19526	+	333	13.1	DUF1108 family protein	Conserved hypothetical protein	83	3×10^{-59}	97.83	CEF82197.1
28	19520-19798	+	279	11	hypothetical protein	hypothetical protein	100	1×10^{-59}	100.00	YP_001429849.1
29	19795-21750	+	1956	75.4	ATPase	hypothetical protein SMAG_02884	100	0.0	100.00	EFG43462.1
30	21752-22672	+	921	34.9	Recombinase	recombinase RecT	100	0.0	100.00	WP_000180600.1
31	22885-23370	+	486	18.3	MBL fold metallo-hydrolase	MBL fold metallo-hydrolase	100	6×10^{-117}	100.00	WP_000123914.1

32	23371-23841	+	471	17.6	single-stranded DNA-binding protein	single-stranded DNA-binding protein	100	5×10^{-109}	100.00	WP_000934759.1
33	23871-24764	+	894	35	replication protein	replication protein	100	0.0	100.00	YP_009113132.1
34	24771-24989	+	219	8.7	hypothetical protein	MULTISPECIES: hypothetical protein	100	3×10^{-46}	100.00	WP_000338528.1
35	24986-25402	+	417	16.3	DNA replication, recombination, and repair protein	DNA replication, recombination, and repair	100	3×10^{-98}	100.00	YP_009113131.1
36	25415-25786	+	372	15	hypothetical protein	hypothetical protein	100	4×10^{-85}	100.00	WP_000101279.1
37	25786-26043	+	256	9.8	MBL fold metallo-hydrolase	phage protein	100	1×10^{-56}	100.00	EVX06805.1
38	26040-26288	+	249	9.9	hypothetical protein	hypothetical protein MQI_02695	100	3×10^{-54}	100.00	EIK11642.1
39	26294-26548	+	255	9.5	hypothetical protein	phage protein	100	2×10^{-52}	100.00	EUS84413.1
40	26545-27081	+	537	20.7	hypothetical protein	dUTP diphosphatase	100	2×10^{-127}	100.00	WP_000185693.1
41	27127-27363	+	237	9.3	hypothetical protein	hypothetical protein	100	1×10^{-46}	100.00	WP_001282077.1
42	27360-27566	+	207	7.82	DUF1381 domain-containing protein	DUF1381 domain-containing protein	100	2×10^{-42}	100.00	WP_000195784.1
43	27563-27736	+	174	6.58	Integrase regulator RinB	Integrase regulator RinB	100	1×10^{-32}	100.00	EFG44756.1
44	27737-27883	+	147	5.72	hypothetical protein	hypothetical protein	100	2×10^{-23}	100.00	WP_000989998.1
45	27907-28329	+	423	16.4	hypothetical protein	DUF1492 domain-containing protein	100	4×10^{-98}	100.00	WP_138271969.1
46	28651-29031	+	381	14.4	DNA-binding protein	DNA-binding protein	100	9×10^{-88}	100.00	WP_031838201.1
47	29015-30280	+	1266	49.1	PBSX family phage terminase, large subunit	PBSX family phage terminase, large subunit	100	0.0	100.00	EZS08983.1
48	30299-31705	+	1407	54.5	phage portal protein	phage portal protein	100	0.0	100.00	WP_078099311.1

49	31644-32624	+	981	37.8	phage head morphogenesis protein	minor capsid protein	100	0.0	100.00	WP_0499949321.1
50	32722-33318	+	597	22.4	hypothetical protein	phage scaffolding protein	100	6×10^{-138}	100.00	WP_015967248.1
51	33339-34163	+	825	29.6	N4-gp56 family major capsid protein	N4-gp56 family major capsid protein	100	0.0	100.00	WP_031838205.1
52	34180-34506	+	327	12.6	Rho termination protein	Rho termination factor N-terminal domain-containing protein	100	4×10^{-70}	100.00	WP_031838206.1
53	34506-34820	+	315	11.9	phage head-tail adapter protein	phage head-tail connector protein	100	7×10^{-71}	100.00	WP_031838207.1
54	34813-35148	+	336	12.9	phage head-tail adapter protein	phage head closure protein	100	2×10^{-75}	100.00	WP_031838208.1
55	35267-35548	+	282	10.2	hypothetical protein	HK97 gp10 family phage protein	100	3×10^{-62}	100.00	WP_072661419.1
56	35561-35998	+	438	16.8	DUF3168 domain-containing protein	DUF3168 domain-containing protein	100	3×10^{-102}	100.00	WP_015967254.1
57	35985-36545	+	561	20.9	hypothetical protein	hypothetical protein	100	7×10^{-133}	100.00	WP_000046067.1
58	36607-37101	+	495	18.7	hypothetical protein	hypothetical protein	100	6×10^{-117}	100.00	WP_000141084.1
59	37122-37463	+	342	13.3	hypothetical protein	hypothetical protein	100	2×10^{-77}	100.00	WP_049949322.1
60	37466-40435	+	2970	106.4	terminase	terminase	100	0.0	100.00	WP_138271971.1
61	38815-39225	-	411	14.2	hypothetical protein	Uncharacterised protein	100	7×10^{-70}	86.03	CAC8570497.1
62	40777-41385	+	609	23.5	phage tail protein	tail protein	100	5×10^{-145}	99.50	ATW69215.1
63	41396-43282	+	1887	70.9	phage minor structural protein	phage tail protein	100	0.0	100.00	WP_113613160.1

Table S2. Summary of similar genomic sequence with phage Henu2

Phage name	Query cover	identity	Accession number	Genome size, bp
Bacteriophage 92	63%	97%	AY954967.1	42431
Bacteriophage 88	62%	97%	AY954966.1	43231
Staphylococcus phage phiETA	51%	97%	AP001553.1	43081
Staphylococcus phage 55-3	60%	96%	KR709303.1	42309
Staphylococcus phage 55-2	60%	96%	KR709302.1	41898
Bacteriophage 55	60%	96%	AY954963.1	41902
Staphylococcus phage phiMR11	62%	96%	AB370268.1	43011
Staphylococcus phage StauST398-5	60%	96%	KC595279.1	43301
Staphylococcus phage StauST398-1	54%	96%	JX013863.1	45242
Staphylococcus phage SA13	59%	96%	JX094501.1	42652
Bacteriophage 71	59%	95%	AY954962.1	43114
Staphylococcus phage UPMK_1	58%	97%	MG543995.1	152788
Staphylococcus phage UPMK_2	57%	97%	MG564297.1	40955
Bacteriophage X2	57%	96%	AY954968.1	43440
Bacteriophage 52A	57%	93%	AY954965.1	41690
Staphylococcus phage 80	56%	93%	DQ908929.1	42140
Staphylococcus phage B166	52%	95%	KP893289.1	42881
Staphylococcus phage phiBU01	19%	99%	KF831354.1	43748
Staphylococcus phage B236	50%	92%	KP893290.1	43228
Staphylococcus phage 3 AJ-2017	20%	99%	KX232515.1	43922

Table S3. List of MIC values of antibiotics

Antibiotic	Classification	Target site	MIC
Tetracycline	Tetracyclines	rRNA	14μg/ml
Cefotaxime	β-lactam	Cell wall	32μg/ml
Linezolid	Oxazolidinone	50S ribosomal	5.5μg/ml
Clarithromycin	Ketolide	50S ribosomal	8.2μg/ml
Ciprofloxacin	Fluoroquinolones	DNA gyrase	2.5μg/ml
H_2O_2	Superoxide	Cell membrane	0.5%

Table S4. One-step growth experiment (Mean ± SD)

Time (min)	0	5	10	15	20	25	30	35	40	45	50	55	60
Log (PFU/mL)	5.67 ± 0.05	6.20 ± 0.04	6.70 ± 0.01	6.90± 0.02	6.94± 0.02	6.96± 0.03	6.96± 0.01	6.98± 0.01	6.96± 0.03	6.97± 0.02	6.97± 0.03	6.95± 0.04	6.96± 0.03

Table S5. pH stability test (Mean ± SD)

PH	2	3	4	5	6	7	8	9	10	11	12
Log (PFU/mL)	0	0	7.37± 0.09	7.43± 0.05	7.37± 0.09	7.57± 0.05	7.60± 0.08	7.07± 0.09	6.30± 0.08	5.30± 0.08	4.10± 0.08

Table S6. Thermal stability test (Mean ± SD)

Temperature (⁰C)	0	10	20	30	40	50	60	70	80	90
Log (PFU/mL)	7.50	7.47± 0.12	7.40± 0.16	7.50± 0.16	7.07± 0.25	6.00± 0.16	4.37± 0.26	0	0	0

Table S7. UV sensitivity of phage Henu2 (Mean ± SD)

Time (min)	0	10	20	30	40	50	60	70	80	90	100	110	120
Log (PFU/mL)	7.43± 0.05	6.97± 0.17	6.60± 0.22	5.67± 0.21	5.10± 0.14	4.07± 0.19	3.57± 0.25	3.27± 0.19	3.13± 0.25	3.00± 0.24	2.87± 0.26	2.87± 0.19	2.87± 0.26

Table S8. Adsorption of phage Henu2 to *S. aureus* (MOI=0.1, Mean ± SD)

Time (min)	0	4	8	12	16	20
**Control**	100.00	75.00± 0.00	45.67± 0.47	28.00± 0.00	13.00± 1.41	2.67± 0.94
**Tetracycline**	100.00	68.33± 0.47	38.33± 0.47	22.33± 2.05	6.00± 0.00	2.00± 0.00
**Cefotaxime**	100.00	66.00± 0.00	33.33± 0.47	17.33± 0.94	4.33± 0.94	0
**Linezolid**	100.00	64.00	29.33± 0.94	13.00± 1.41	2.00	0
**Clarithromycin**	100.00	61.33± 0.94	25.33± 0.47	6.67± 1.89	2.33± 0.47	0
**Ciprofloxacin**	100.00	74.67± 0.47	43.67± 0.47	24.33± 1.70	8.00	2.67± 0.94
**H_2O_2**	100.00	73.00	40.67± 0.94	24.33± 1.70	7.00	2.00

Table S9. Burst sizes measured in the presence of antibiotics inducing PAS (Mean ± SD)

Group	Control	LZD	CLM	CTX	TET	CIP	H2O2
Burst sizes	7.80± 0.57	28.80± 1.49	16.60± 0.57	18.30± 0.59	9.90± 0.33	9.90± 0.67	19.50± 0.49

Table S10. Killing effect of phage Henu2 on *S. aureus* N315 logarithmic phase cells (Mean ± SD)

Time (h)	2	4	6	8	10	12	14
**Control**	6.80	7.13± 0.05	7.63± 0.12	8.20± 0.08	8.63± 0.05	8.87± 0.05	9.03± 0.12
**Linezolid**	6.80	6.60± 0.08	6.90± 0.08	7.67± 0.09	8.17± 0.12	8.50± 0.00	8.63± 0.05
**Clarithromycin**	6.80	6.93± 0.12	7.40± 0.08	8.07± 0.12	8.70± 0.08	8.50± 0.08	8.83± 0.09
**Cefotoxime**	6.80	7.07± 0.09	7.53± 0.05	7.83± 0.05	8.27± 0.05	8.63± 0.09	8.83± 0.05
**Tetracycline**	6.80	6.50	6.80	7.03± 0.05	7.40± 0.08	7.53± 0.05	7.83± 0.05
**Ciprofloxacine**	6.80	6.83± 0.09	7.17± 0.12	7.67± 0.12	7.87± 0.05	8.27± 0.05	8.50± 0.08
**H_2O_2**	6.80	7.07± 0.09	7.47± 0.05	7.83± 0.05	8.17± 0.05	8.40± 0.08	8.67± 0.05
**Henu2**	6.80	6.47± 0.05	6.17± 0.05	6.10± 0.08	5.83± 0.12	5.80± 0.08	6.17± 0.12
**Linezoid+Henu2**	6.80	6.10± 0.08	4.87± 0.26	4.00± 0.16	3.37± 0.12	2.77± 0.21	2.27± 0.17
**Clarithromycin+Henu2**	6.80	6.27± 0.05	5.23± 0.19	4.57± 0.17	3.90± 0.08	3.60± 0.08	3.33± 0.24
**Cefotoxime+Henu2**	6.80	6.30± 0.16	5.30± 0.08	4.50± 0.24	3.50± 0.24	3.27± 0.21	3.10± 0.22
**Ciprofloxacine+Henu2**	6.80	6.27± 0.05	5.70± 0.22	5.30± 0.08	4.80± 0.14	4.40± 0.14	4.00± 0.16
**H2O2+Henu2**	6.80	6.27± 0.05	5.33± 0.12	4.70± 0.08	3.67± 0.34	3.40± 0.08	2.87± 0.33
**Tetracycline+Henu2**	6.80	6.23± 0.17	5.23± 0.21	4.43± 0.12	3.73± 0.12	3.27± 0.09	2.70± 0.16
